# A New Lighting System for Surgical Vision Optimization in Barbed Pharyngoplasty for OSA

**DOI:** 10.3390/jpm13091320

**Published:** 2023-08-28

**Authors:** Lorenzo Sabatino, Antonio Moffa, Francesco Iafrati, Simone Di Giovanni, Luigi De Benedetto, Lucrezia Giorgi, Peter Baptista, Claudio Vicini, Andrea De Vito, Manuele Casale

**Affiliations:** 1 Integrated Therapies in Otolaryngology, Fondazione Policlinico Universitario Campus Bio-Medico, 00128 Rome, Italy; 2 School of Medicine, Università Campus Bio-Medico di Roma, 00128 Rome, Italy; 3 Unit of Measurements and Biomedical Instrumentation, Università Campus Bio-Medico di Roma, 00128 Rome, Italy; 4 ENT Department, Al Zahra Private Hospital Dubai, Dubai 23614, United Arab Emirates; 5 Department of Otorhinolaryngology, Clinica Universidad de Navarra, 31008 Pamplona, Spain; 6 ENT Department, Morgagni Pierantoni Hospital, 47121 Forli, Italy; 7 ENT Department, University of Ferrara, 44121 Ferrara, Italy; 8 Otolaryngology and Head-Neck Surgery Unit, Department of Surgery, Ravenna & Lugo Hospitals, Health Local Agency of Romagna, 48121 Ravenna, Italy

**Keywords:** barbed pharyngoplasty, lighting system, surgical vision, oral cavity, KLARO^TM^

## Abstract

Obstructive sleep apnea (OSA) surgery is now a viable solution in selected patients and the “remodeling” palatopharyngeal surgery is the most common one. Recently, it has become less invasive with the introduction of barbed sutures (BS). An optimization of surgical techniques is represented by barbed pharyngoplasty (BP), which requires surgical precision and needs efficient and precise oropharyngeal visualization. Consequently, the lighting system is of pivotal importance in BP. The aim of this work is to describe the first experience on the use of a new lighting system, called KLARO^TM^ in BP for OSA. We evaluated the KLARO™ system in 15 consecutives BP for OSA in comparison with conventional headlamp illumination. The visualization of palatopharyngeal muscle in the bottom of the tonsillar fossa, entry and exit needle, such as needle tip, were statistically better with KLARO^TM^ than headlamp illumination for both the surgeon and resident (*p* < 0.05). No significant differences for the visualization of the posterior pharyngeal wall and uvula were reported. The KLARO^TM^ lighting system allows a satisfied illumination of oral cavity and oropharynx in the majority of cases. We encourage the use of KLARO^TM^ not only in BP for OSA, but in all oral and pharyngeal surgeries, including tonsillectomy and oncological surgery.

## 1. Introduction

Obstructive sleep apnea (OSA) is a growing health concern involving about one billion people worldwide; it is characterized by episodes of vibration and collapse of upper airways during sleep, resulting in noise production (snoring), airflow decreasing (hypopnea) or cessation (apnea), oxygen desaturations, fragmentation of sleep, and daytime sleepiness [1]. The first-line treatments that are frequently employed in moderate and severe OSA are continuous positive airway pressure (CPAP) and/or a mandibular advancement device (MAD) [2,3]; however, failure in long-term adherence to both treatments was reported in 25–50% of cases [4]. These factors have led to significant advances in OSA and snoring surgery management over the past few years. In patients who do not tolerate or do not have good results with first lines treatments, OSA surgery is now a viable solution, thanks to newer, less invasive, or morbid treatments that also increase patient compliance [5]. Among the surgical procedures for OSA treatment, palatopharyngeal surgery is one the most commonly performed [6]. To preserve pharyngeal function and improve breathing space, oropharyngeal surgery for OSA has progressed from a significant removal of “redundant” soft tissue (resection techniques) to less invasive reconstruction techniques.

To reduce surgical procedures’ invasiveness and increase oropharyngeal stiffness in recent years, Vicini et al. [7] made popular a new suturing technique known as barbed sutures pharyngoplasty (BP), which allows a knot-free tissue closure and a uniform distribution of tensile closure force [8].

BP requires more surgical precision from the surgeon, following precise vectors in a submucosal plane during the procedure [9].

Generally, the surgeon illuminates the operating field with his conventional headlight (photophore), but often this is not enough to achieve an optimal and uniform illumination of the entire surgical area. In addition, due to the depth of the oral cavity and often the narrow mouth of patients, it is difficult for trainees to correctly view all the steps of surgical procedures performed by the first surgeon.

In transoral surgical procedures the surgeon is often forced to assume uncomfortable positions for a long time [10], with difficult exposure of the operating field and poor sharing of the procedure with assistants and trainees [11].

**Figure 1:**
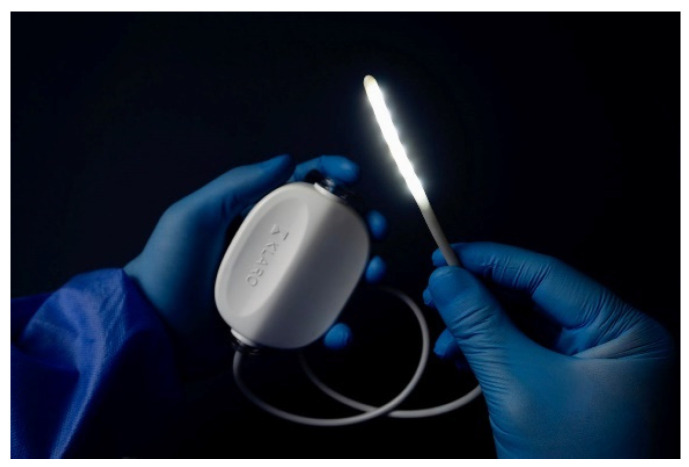
KLARO^TM^ device.

A new device called KLARO^TM^ (Figure 1) has been recently introduced to the market for the illumination of anatomical cavities during surgical operations. The KLARO^TM^ (Vivo Surgical Private Limited, Singapore) is a sterile and disposable surgical lighting device for deep cavities.

This work aims to describe our experience using KLARO^TM^ as an additional illumination tool, especially in BP for OSA, to facilitate surgeons’ work and residents’ learning curve.

## 2. Materials and Methods

### 2.1. Patients’ Selection

We used the KLARO™ device in 15 consecutives patients (13 males, 2 females; median age 49.1 ± 9.87 years old) affected by moderate-severe obstructive OSA, who underwent BP at the Unit of Integrated Therapies in Otolaryngology at Campus Bio-Medico University Hospital Foundation from January to May 2022.

The inclusion criteria were OSA patients older than 18 years old, with a confirmed circular palatal collapse at drug-induced sleep endoscopy assessment, who refused or did not tolerate nasal CPAP therapy as first-line treatment. Furthermore, all the patients enrolled in the study showed good nasal patency, small tonsils (tonsil size 1 and 2 according to Friedman Staging System), BMI less than 30 kg/m^2^, and ASA < 2. Patients with age more than 70-year-old, with severe medical comorbidities, Mallampati grade IV were excluded from the study.

### 2.2. Surgical Procedure

The surgical technique used was Alianza Barbed Pharyngoplasty. It is performed under general anesthesia with orotracheal intubation; the patient is placed in supine position, and a Boyle–Davis mouth gag is used to expose the oropharynx.

It uses barbed absorbable sutures that allow suspending palato-pharyngeal structures to anatomical non-collapsible landmarks (posterior nasal spine, pterigoideal hamulus, pterigomandibular raphe) [12].

We used two interlaced unidirectional barbed threads (Medtronic V-Loc™ 180, size 2–0 or 3–0, length 30 cm, mounted on taper-pointed 26 mm semicircular needle, absorption in 180 days, tensile strength 65% at 21 days) to obtain a bidirectional suture: each needle is passed in the looped-end of the other suture, then gentle traction is applied to tighten a “flat knot”.

To prevent the two looped ends being left protruding in the oral cavity, a tiny (3 mm) incision in the mucosa over the Posterior Nasal Spine (PNS) is made to sink them in [8].

### 2.3. The Illumination Device

The KLARO™ comprises of a fully flexible 4.6 mm diameter LED light strip and a clip-like driver unit. The LED light strip can be safely placed deep inside an open surgical cavity. It is freely bendable and provides variable angles of wide illumination of over 180°. The driver unit can be fastened onto surgical drapes during use. The entire device is a single-use disposable that maintains a working temperature of below 38 °C over a 4 h lifespan, ensuring sterility and zero tissue-burns. KLARO™ is, therefore, appropriate for most open surgery applications.

The KLARO™ is registered and approved in several international territories, including the US FDA, European Union’s (EU) CE Mark, and Singapore’s Health Science Authority (HSA).

### 2.4. Mounting the KLARO^TM^ Device on Tongue Retractor

**Figure 2:**
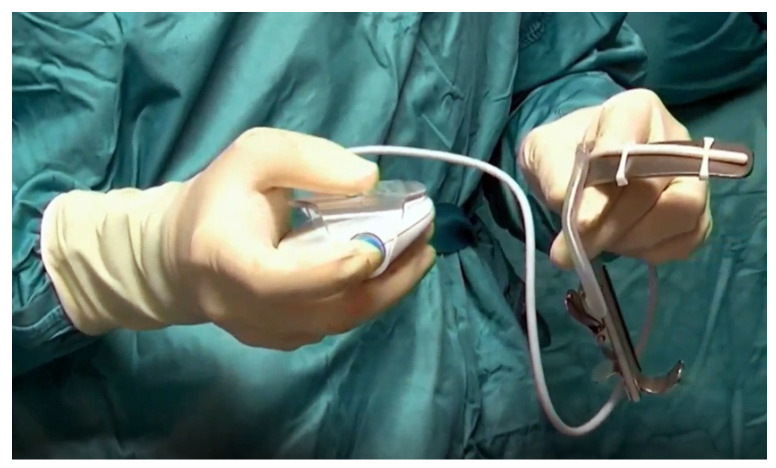
KLARO^TM^ device applied on the tongue retractor.

The device is provided with 4 Retractor Loops and 2 Mini Retractor Loops that enable the user to fasten the KLARO™ LED strip easily and quickly onto most surgical retractors in the market (Figure 2).

### 2.5. Study Design

**Figure 3:**
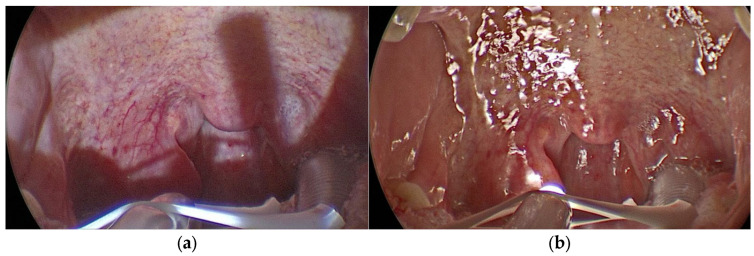
(**a**) Oropharynx lighting with headlamp; (**b**) oropharynx lighting with KLARO^TM^.

On each patient, the procedure was performed by an expert surgeon: on one half of the palate with the headlamp and the other half with KLARO^TM^ illumination system; the residents attended the surgical procedures, which were filmed with a 70° rigid endoscope and transmitted on the screen (Figure 3a,b).

For the illumination system evaluation, we asked both the first surgeon and residents about the quality of vision of critical structures (Uvula, Posterior Pharyngeal Wall, Palatopharyngeus muscle, penetration and exit point of the needle, and Needle tip), grading it with a score from 1 to 3 (1 = poor vision, 2 = moderate vision, 3 = good vision). 

### 2.6. Statistical Analysis

The comparison between KLARO^TM^ and headlamp was made using a Wilcoxon’s signed rank sum test. Statistical significance was set at a *p*-value < 0.05, and the data were analyzed using R version 4.1.2 (R Foundation for Statistical Computing, Vienna, Austria).

## 3. Results

### 3.1. Intraoperative Quality of Visualization of Anatomical Structures According to the Surgeon: Headlamp vs. KLARO^TM^

The quality of visualization was statistically significantly better with KLARO^TM^ versus headlamp identifying the palatopharyngeal muscle fibers at the bottom of the tonsillar fossa, allowing us to perform delicate maneuvers such as entry and exit with the needle at the same point. No significant differences were reported for the visualization of the posterior pharyngeal wall and uvula (Table 1).

### 3.2. Intraoperative Quality of Visualization of Anatomical Structures According to the Residents: Headlamp vs. KLARO^TM^

The quality of visualization on the screen was statistically significantly better with KLARO^TM^ versus the headlamp to identify the fibers of the palatopharyngeal muscle and to recognize the entrance and exit point of the sutures and the needle tip (Table 2). The uniformity of vision and the absence of shadows on the screen during the use of KLARO^TM^ made the image more uniform and enjoyable (Figure 3a,b). Also, the residents did not report significant differences for the visualization of the posterior pharyngeal wall and uvula.

## 4. Discussion

Palato-oropharyngeal remodeling surgery has become central in OSA surgical management, and barbed pharyngoplasties are innovative techniques in this field. Barbed pharyngoplasties allow the surgeon to lift and stiffen the soft palate and lateral pharyngeal walls to counter their hyper-collapsibility during sleep while preserving their anatomical and functional integrity.

The main limitations deriving from the execution of BP derive from the knowledge of barbed sutures and the narrow field in which it operates. The oral cavity is a structure that is not always easily attacked surgically, especially in OSA patients characterized by a reduced buccal opening and a hypertrophic tongue. Furthermore, the mouth is a space with reduced light, another factor that can limit the visualization of the structures.

Very often the surgeon illuminates the oral cavity with his conventional headlight (photophore), but often it is not enough to extensively illuminate the surgical site.

The KLARO^TM^ lighting system proved in this preliminary study, it is possible to light up all the structures in the oral cavity, providing a better visualization than conventional methods for both surgeons and observers. All major surgical landmarks can be illuminated and visualized during barbed pharyngoplasty.

The limitations of this study include a small number of participants who used a KLARO^TM^ lighting system to a single snoring and OSA center. This increases the potential of a response bias for quality of visualization of anatomical structure appraisal. We hope that in the future it will be used in other centers that perform barbed pharyngoplasty and for other kinds of oral surgery including oncological surgery. It could be very difficult to perform comparative studies in order to evaluate surgical outcomes and patients’ experiences. Surely, as a future prospective, we will evaluate both of these aspects.

## 5. Conclusions

The KLARO^TM^ lighting system is a good and comfortable illumination device for oropharyngeal surgery that improves the surgeon’s ergonomics, providing a better visualization for observers learning of surgical procedures; the uniform illumination device allows us to identify the anatomical landmarks performing barbed pharyngoplasty accurately.

The KLARO^TM^ lighting system is easily adaptable to the retractors thanks to its flexibility and adequate hooking systems, even if sometimes, especially in patients with Mallampati IV and/or reduced mouth opening can be cumbersome. Our experience with KLARO^TM^ is focused on barbed pharyngoplasty for OSA, but we encourage the use of KLARO^TM^ in all oral and pharyngeal surgeries, including tonsillectomy and oncological surgery.

## Figures and Tables

**Table 1:** Intraoperative quality of visualization of anatomical structures according to the surgeon: headlamp vs. KLARO^TM^.

	Headlamp (n° 15)			KLARO^TM^ (n° 15)			*p*-Value
	Good	Moderate	Poor	Good	Moderate	Poor	
Uvula	10	5	0	13	2	0	0.08
Posterior Pharyngeal Wall	9	6	0	11	4	0	0.16
Palatopharyngeus muscle	6	4	5	8	4	3	0.04
In and exit point	6	7	2	9	5	1	0.04
Needle tip	7	5	3	10	4	1	0.02

**Table 2:** Intraoperative quality of visualization of anatomical structures according to the residents: headlamp vs. KLARO^TM^.

	Headlamp (n° 15)			KLARO^TM^ (n° 15)			*p*-Value
	Good	Moderate	Poor	Good	Moderate	Poor	
Uvula	8	5	2	9	5	1	0.16
Posterior Pharyngeal Wall	9	5	1	11	3	1	0.16
Palatopharyngeus muscle	3	8	4	5	8	2	0.04
In and exit point	6	6	3	8	6	1	0.04
Needle tip	5	7	3	8	6	1	0.02

## Data Availability

All data generated or analyzed during this study are included in this published article.

## References

[1] Mantovani M., Minetti A., Torretta S., Pincherle A., Tassone G., Pignataro L. (2013). The “Barbed Roman Blinds” technique: A step forward. Acta Otorhinolaryngol. Ital..

[2] Patil S.P., Ayappa I.A., Caples S.M., Kimoff R.J., Patel S.R., Harrod C.G. (2019). Treatment of Adult Obstructive Sleep Apnea with Positive Airway Pressure: An American Academy of Sleep Medicine Clinical Practice Guideline. J. Clin. Sleep Med..

[3] Ramar K., Dort L.C., Katz S.G., Lettieri C.J., Harrod C.G., Thomas S.M., Chervin R.D. (2015). Clinical Practice Guideline for the Treatment of Obstructive Sleep Apnea and Snoring with Oral Appliance Therapy: An Update for 2015. J. Clin. Sleep Med..

[4] Chiu F.H., Chen C.Y., Lee J.C., Hsu Y.S. (2021). Effect of Modified Uvulopalatopharyngoplasty without Tonsillectomy on Obstructive Sleep Apnea: Polysomnographic Outcome and Correlation with Drug-Induced Sleep Endoscopy. Nat. Sci. Sleep.

[5] Kent D., Stanley J., Aurora R.N., Levine C., Gottlieb D.J., Spann M.D., Torre C.A., Green K., Harrod C.G. (2021). Referral of adults with obstructive sleep apnea for surgical consultation: An American Academy of Sleep Medicine clinical practice guideline. J. Clin. Sleep Med..

[6] Sheen D., Abdulateef S. (2021). Uvulopalatopharyngoplasty. Oral. Maxillofac. Surg. Clin. North Am..

[7] Vicini C., Meccariello G., Montevecchi F., De Vito A., Frassineti S., Gobbi R., Pelucchi S., Iannella G., Magliulo G., Cammaroto G. (2020). Effectiveness of barbed repositioning pharyngoplasty for the treatment of obstructive sleep apnea (OSA): A prospective randomized trial. Sleep Breath..

[8] Casale M., Moffa A., Giorgi L., Sabatino L., Pierri M., Lugo R., Baptista P., Rinaldi V. (2022). No-cutting remodelling intra-pharyngeal surgery can avoid CPAP in selected OSA patients: Myth or reality?. Eur. Arch. Otorhinolaryngol..

[9] Gulotta G., Iannella G., Meccariello G., Cammaroto G., Visconti I.C., de Vincentiis M., Greco A., Pelucchi S., Magliulo G., Ruoppolo G. (2021). Barbed suture Extrusion and Exposure in palatoplasty for OSA: What does it mean?. Am. J. Otolaryngol..

[10] Sahni D. (2015). Is there an Increased Incidence of Cervical Degenerative Disease in Surgeons who use Loupes and a Headlight?. J. Spine Surg..

[11] Festa B.M., Zuppardo J., Costantino A., Ferreli F., Spriano G., Mercante G., De Virgilio A. (2022). High-definition 3D exoscope-assisted tonsillectomy. Am. J. Otolaryngol..

[12] Moffa A., Rinaldi V., Mantovani M., Pierri M., Fiore V., Costantino A., Pignataro L., Baptista P., Cassano M., Casale M. (2020). Different barbed pharyngoplasty techniques for retropalatal collapse in obstructive sleep apnea patients: A systematic review. Sleep Breath..

